# Phase morphology, mechanical, and thermal properties of fiber-reinforced thermoplastic elastomer: Effects of blend composition and compatibilization

**DOI:** 10.1177/07316844211051749

**Published:** 2021-10-22

**Authors:** Ali Fazli, Denis Rodrigue

**Affiliations:** 1 Department of Chemical Engineering, 98637 Université Laval, Quebec, QC, Canada

**Keywords:** Recycling, tire rubber, tire fiber, thermoplastic elastomers, composites, compatibilization

## Abstract

In this work, recycled high density polyethylene (rHDPE) was compounded with regenerated tire rubber (RR) (35–80 wt.%) and reinforced with recycled tire textile fiber (RTF) (20 wt.%) as a first step. The materials were compounded by melt extrusion, injection molded, and characterized in terms of morphological, mechanical, physical, and thermal properties. Although, replacement of the rubber phase with RTF compensated for tensile/flexural moduli losses of rHDPE/RR/RTF blends because of the more rigid nature of fibers increasing the composites stiffness, the impact strength substantially decreased. So, a new approach is proposed for impact modification by adding a blend of maleic anhydride grafted polyethylene (MAPE)/RR (70/30) into a fiber-reinforced rubberized composite. As in this case, a more homogeneous distribution of the fillers was observed due to better compatibility between MAPE, rHDPE, and RR. The tensile properties were improved as the elongation at break increased up to 173% because of better interfacial adhesion. Impact modification of the resulting thermoplastic elastomer (TPE) composites based on rHDPE/(RR/MAPE)/RTF was successfully performed (improved toughness by 60%) via encapsulation of the rubber phase by MAPE forming a thick/soft interphase decreasing interfacial stress concentration slowing down fracture. Finally, the thermal stability of rubberized fiber-reinforced TPE also revealed the positive effect of MAPE addition on molecular entanglements and strong bonding yielding lower weight loss, while the microstructure and crystallinity degree did not significantly change up to 60 wt.% RR/MAPE (70/30).

## Introduction

Recycling the increasing amount of waste tires across the globe as hazardous materials accumulating in landfills is a worldwide environmental concern since their natural decomposition is estimated to be over 600 years.^1^ Presently, end-of-life (EOF) tire rubber and tire textile fibers are buried or burned as tire-derived fuels releasing toxic gases.^2^ Therefore, alternative environmentally friendly and added-value uses for these large amounts of wastes are required to be developed. Compared to virgin rubbers, using recycled rubber (mainly obtained from waste tires) benefits from lower cost (less use of raw materials), environmental friendliness, and simpler processing conditions (there is no need for dynamic vulcanization of the elastomer phase).^3^ The most common option in terms of rubber recycling is to combine waste tire rubber with thermoplastic resins to develop fully recycled compounds called thermoplastic elastomers (TPE) with reduced materials costs and enhanced performance/processability of plastics and rubbers.^4^ However, the crosslinked network of ground tire rubber (GTR) does not have enough molecular freedom to entangle with the matrix macromolecules resulting in low compatibility and weak interfacial adhesion which is the origin of poor mechanical properties and low durability of these compounds.^5,6^ In general, GTR introduction serving as stress concentration points around the rubber clusters might result in multiple micro-void formations at the interface facilitating fracture by lowering the absorbed energy before break-up.^7^

But waste tire rubber can be subjected to a regeneration process by partially breaking down the crosslinked structure via C-S and/or S-S bonds scission with limited hydrocarbon backbone chains rupture. Therefore, the soluble fraction of regenerated tire rubber (RR) can generate strong interactions between the TPE phases.^8^ However, it is difficult to obtain a high sol fraction with acceptable molecular weight (MW) without scission of the main rubber chains resulting in a MW drop coupled with a loss of mechanical strength.^4^

One way of overcoming this problem is the use of short fibers inducing good strength and stiffness.^9,10^ Fiber-reinforced TPE has been shown to have good mechanical properties leading to a growing interest due to the lower density of these reinforcements combined with lower cost, renewability, and environmentally friendly source of several fibers.^3,11,12^ The efficiency of short fiber reinforcements depends on the fiber type, aspect ratio, concentration, orientation, and distribution after mixing, as well as the level of adhesion between the fiber and the matrix.^13^ But the low affinity of short fiber and crosslinked rubber particles toward several polymer matrices contribute to high surface energy and phase incompatibility leading to poor elongation at beak and impact strength due to insufficient interfacial bonding.^14,15^ Once good adhesion is obtained, the incorporation of fibers can lead to increased tensile and flexural properties of the composites.^7^ For example, Kakroodi et al.^16^ observed that the tensile modulus of recycled polypropylene (rPP)/GTR (80/20) blends was improved by 25% (from 320 to 400 MPa) after the incorporation of 20 wt.% birch wood flour. However, introducing high amounts of fibers (serve as stress concentrators) led to interfacial voids creating structural defects due to fiber–fiber interactions and poor dispersion, thus decreasing impact resistance (toughness).^17^ To solve this problem, the addition of elastomers is the most common method to increase the impact strength (toughness) increasing the amount of energy absorbed before rupture.^18^ To this end, several copolymers, such as ethylene-propylene-diene monomer (EPDM),^19^ styrene-butadiene-styrene (SBS),^20^ and styrene-ethylene-butylene-styrene,^21^ have been proposed for impact modification. Lima et al.^22^ claimed that EPDM tends to coat the recycled tire particles surface providing a soft interface improving compatibility with PP. The results showed that the impact strength of PP/EPDM/GTR (70/15/15) increased by 65% (from 2.9 to 4.8 kJ/m^2^) compared to PP/GTR (70/30).

The addition of maleated polyolefins (interfacial modifiers) was also shown to be very effective by forming a strong interface between the rubber particles and thermoplastic matrices via selective localization at the interfacial area between immiscible polymer blends leading to improved physical compatibility (higher interfacial adhesion) resulting in higher tensile properties.^23–25^ For example, the addition of 10 wt.% of maleic anhydride grafted polyethylene (MAPE) into high density polyethylene (HDPE) filled with 30 wt.% of reclaimed rubber increased the elongation at break by 10% (from 125 to 138%). This improvement was related to chemical bonds formed between the maleic anhydride group of MAPE and unsaturated C = C bonds on the rubber surface.^23^ Tensile elongation at break helps to determine the compatibility and homogeneity of TPE blends, while elongation at break of recycled TPE are lower than virgin compounds because of contamination and impurities (crazing points), as well as degradation of recycled materials (mechanical and thermal stresses) during their service life, grinding, and regeneration.^26,27^

Although a large body of literature is available on recycled tire rubber, very few studies investigated the potential of recycled tire fibers (RTF) for TPE reinforcement.^28,29^ Hence, this work investigates the effect of both recycled tire rubber and fiber contents on the properties of TPE composites with a focus on the structure–property relationships. The effect of reinforcement type and content on the phase morphology, as well as mechanical and thermal properties, especially blend toughening, was thoroughly investigated. In particular, a new approach is proposed for impact modification by using a RR/MAPE masterbatch into a fiber-reinforced rubberized composite. The results also show how the encapsulation of the rubber phase by MAPE can further improve the physical compatibility (higher interfacial adhesion) and the fracture resistance of a fiber-reinforced system combined with improved stiffness.

## Experimental

### Materials

**Figure 1.**
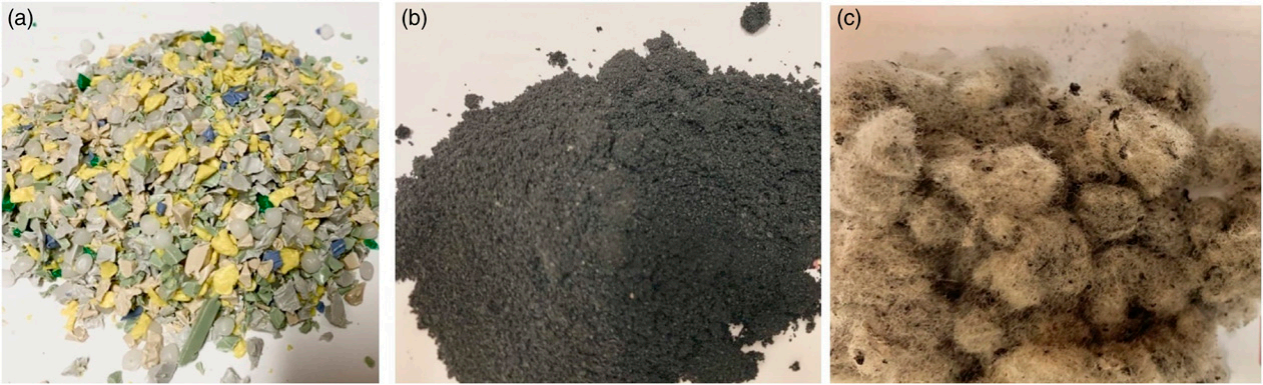
General view of: (a) rHDPE flakes, (b) RR particles, and (C) RTF as received. rHDPE: recycled high density polyethylene; RTF: recycled tire textile fiber.

Post-consumer rHDPE in flakes coming from recycled solid HDPE bottles was used as thermoplastic matrix (Figure 1(a)). Recycled rubber particles (RR) from regenerated car tire as rubber phase and RTF as reinforcement fibers were used without modification (Figure 1(b) and (c)). The MAPE was used as coupling agent to compatibilize fiber-reinforced rubberized composites. Table 1 presents an overview of the materials used for this study.

**Table 1.** Specifications and properties of the materials used.

Material	rHDPE	RR	RTF	MAPE
Commercial name	—	PI3.1.C	—	Epolene C-26
Producer/supplier	Service de consultation sinclair (Drummondville, Canada)	Phoenix innovation technologies (Montreal, Canada)	Quebec transloc (Lévis, Canada)	Westlake chemical corp (TX, USA)
Density (ASTM D2856^30^)	0.986 g/cm^3^	1.184 g/cm^3^	1.268 g/cm^3^	0.920 g/cm^3^
MFI (190°C and 2.16 kg; ASTM D1238^31^)	6.7 g/10 min	—	—	8 g/10 min
Form (appearance)	Flakes	Powders	Fibers/Fluffy	Pellets
Remarks	Melting point of 127.5°C (ASTM D3418^32^)	Average particle size of 500 μm	—	MW of 65 kg/mol, and acid number of 8 mg KOH/g

rHDPE: recycled high density polyethylene; RTF: recycled tire textile fiber.

### Processing

A co-rotating twin-screw extruder Leistritz ZSE-27 with a L/D ratio of 40 and 10 heating zones (die diameter of 2.7 mm) was used for melt blending of samples. The melt extrusion temperature was set at 175°C for all zones to limit RR degradation, while the screw speed was set at 120 r/min. The overall flow rate was 4 kg/h for all the blends to prevent high motor torque and die pressure associated with the high viscosity of RR compounds. The materials were cooled in a water bath and then pelletized using a model 304 pelletizer (Conair, Stanford, USA) followed by drying for 6 h in an oven at 70°C to eliminate any residual water for further processing (injection molding).

#### Composites without compatibilizer

**Figure 2.**
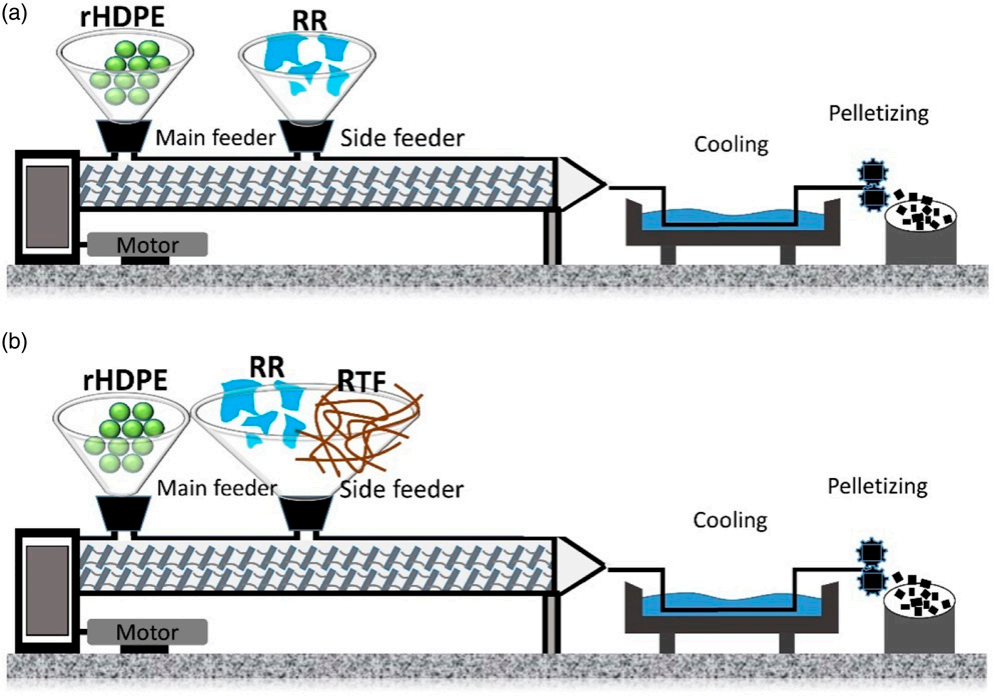
Melt extrusion of: (a) rHDPE/RR and (b) rHDPE/RR/RTF samples. rHDPE: recycled high density polyethylene; RTF: recycled tire textile fiber.

Different rHDPE-based composites with fillers (RR or RR/RTF) were produced with various compositions as presented in Table 2. As shown in Figure 2(a), the rHDPE pellets were introduced through the main feeder (zone 1), while the RR particles (35, 50, 65, and 80 wt.%) were introduced via a side-stuffer located in zone 4 of the extruder to limit thermal degradation. Then, different concentrations of RR particles (15, 30, 45, and 60 wt.%) were dry-blended with RTF (20 wt.%) after being oven-dried at 70°C for 12 h. Again, the rHDPE was fed to the extruder in the first zone (main feed), while the RR/RTF mixtures were fed via the side feeder (zone 4). The processing temperature was fixed at 175°C with a screw speed of 120 r/min and a flow rate of 4 kg/h. All the extrudates were cooled in a water bath before pelletizing (Figure 2(b)).

**Table 2.** List of the compositions investigated (% wt.).

Sample	rHDPE	RR	RTF	Masterbatch RR/MAPE (70/30)
RHD	100	—	—	—
R35	65	35	—	—
R50	50	50	—	—
R65	35	65	—	—
R80	20	80	—	—
R15F	65	15	20	—
R30F	50	30	20	—
R45F	35	45	20	—
R60F	20	60	20	—
R15F*	65	—	20	15
R30F*	50	—	20	30
R45F*	35	—	20	45
R60F*	20	—	20	60

rHDPE: recycled high density polyethylene; RTF: recycled tire textile fiber.

#### Composites with compatibilizer

**Figure 3.**
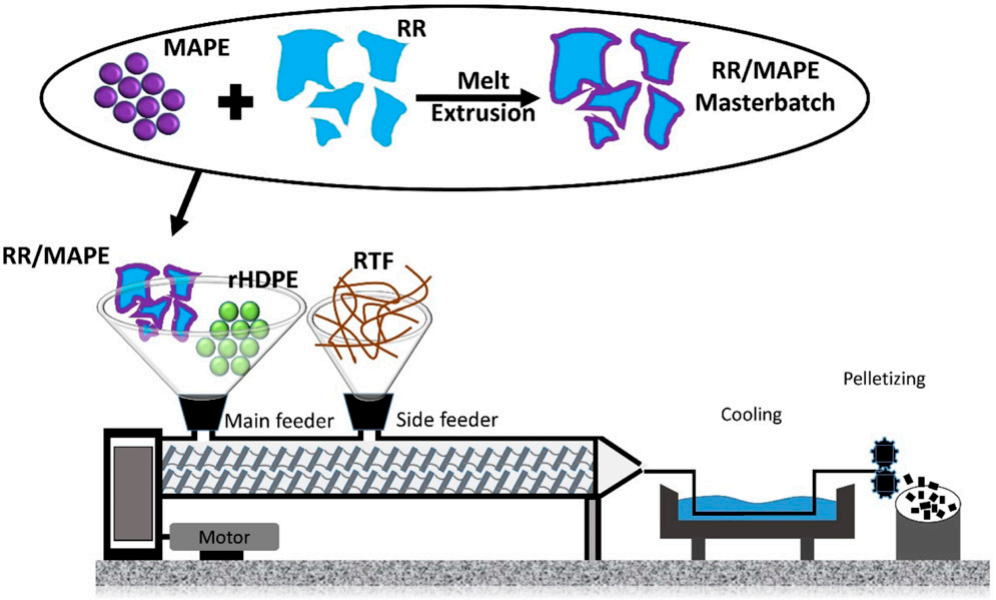
Melt extrusion steps for the different rHDPE/(RR/MAPE)/RTF samples. rHDPE: recycled high density polyethylene; RTF: recycled tire textile fiber.

As illustrated in Figure 3, RR/MAPE masterbatches were produced by melt blending of RR particles (70 wt.%) with MAPE pellets (30 wt.%) to get good surface coverage. In this case, the MAPE pellets were fed to the extruder in the first zone (main feed), while RR particles were fed via the side feeder (zone 4). The processing conditions were fixed at a temperature of 175°C, a screw speed of 120 r/min and a flow rate of 4 kg/h. Again, the materials were cooled in a water bath and pelletized. Then, these pellets (RR/MAPE masterbatch) were introduced in the main feeder at different concentrations (15, 30, 45, and 60 wt.%) along with rHDPE (65, 50, 35, and 20 wt.%) in a second extrusion step, while the RTF (20 wt.%) was introduced via the side-stuffer located at zone 4. All the formulations with codes are presented in Table 2. After drying, the final samples were produced on a PN60 (Nissei, Japan) injection molding machine. The temperature profile was set as 180–170-170-160°C (nozzle, front, middle, and rear). The mold had four cavities to directly produce the standard geometries for characterization. The injection pressure was adjusted (45–55 MPa) depending on the compound viscosity, while the mold temperature was fixed at 30°C.

### Characterization

#### Morphology

An Inspect F50 scanning electron microscope (SEM) (FEI, Hillsboro, OR, USA) was used at 15 kV to take micrographs of the raw materials and observe the quality of the interfacial adhesion/dispersion in the blends. The samples were cryogenically fractured in liquid nitrogen and the surfaces were coated with gold/palladium to be observed at different magnifications. RR and RTF were also investigated by energy dispersive spectroscopy (EDS) using the same device to identify impurities (contamination).

#### Mechanical testing

Tensile tests were conducted at room temperature according to ASTM D638-14^33^ using a 500 N load cell and a 10 mm/min crosshead speed on an Instron (Instron, Norwood, MA, USA) universal mechanical tester model 5565. At least 5 specimens (type IV) with 3 mm thickness were used for each formulation. The averaged values of tensile strength (σ_Y_), Young’s modulus (E), and elongation at break (ε_b_) are reported with standard deviations.

Flexural tests were done on an Instron (Instron, Norwood, MA, USA) model 5565 with a 50 N load cell according to ASTM D790-10^34^ at room temperature. Rectangular specimens with dimensions of 60 × 12.7 mm^2^ were tested with 5 repetitions for each sample in a three-point bending mode (span length of 60 mm) at a crosshead speed of 2 mm/min.

Notched Charpy impact strength was measured on a Tinius Olsen (Horsham PA, USA) model 104 at room temperature according to ASTM D256-10.^35^ At least 10 specimens with dimensions of 60x12.7 mm^2^ were used for each compound. Before testing, all the samples were automatically V-notched on a Dynisco (Franklin, MA, USA) model ASN 120 m sample notcher 24 h before testing.

#### Physical properties

Hardness (Shore D) was determined by a model 307L durometer (PTC Instruments, Boston, MA, USA) with 10 measurements for each sample.

Density was determined by a gas (nitrogen) pycnometer Ultrapyc 1200e (Quantachrome Instruments, Boynton Beach, FL, USA). Each measure was repeated three times for each sample.

#### Thermogravimetric analysis

Thermal stability of the raw materials and the compounds were investigated via thermogravimetric analysis (TGA) on a Q5000 IR (TA Instruments, New Castle, DE, USA) with a heating rate of 10°C/min from 50 to 850°C. The tests were performed in nitrogen and air atmospheres to evaluate both thermal and oxidative resistance of the materials.

#### Differential scanning calorimetry

The melting and crystallization behaviors of the samples were examined on a differential scanning calorimetry (DSC7) (Perkin Elmer, USA). About 5–10 mg of sample was placed in an aluminum pan and the test was performed by heating from 50 to 200°C at 10 °C/min under a nitrogen atmosphere followed by cooling back to 50°C at 10°C/min. The maximum of the endothermic peak, the maximum of the exothermic peak, and the area under the endothermic peak were used for evaluation of the melting temperature (T_m_), crystallization temperature (T_c_), and enthalpy of fusion (ΔH_m_) of the samples, respectively. Also, the matrix crystallinity degree (X) was calculated as

(1) $$X=\frac{\Delta H_{m}}{\left(1-\phi \right)\Delta H_{m0}}100$$

where φ is the total weight fraction of filler (RR + RTF) in the blend and ΔH_m0_ is the melting enthalpy of 100% crystalline HDPE (285.8 J/g).^23^

## Results and discussion

### Morphological characterization

**Figure 4.**
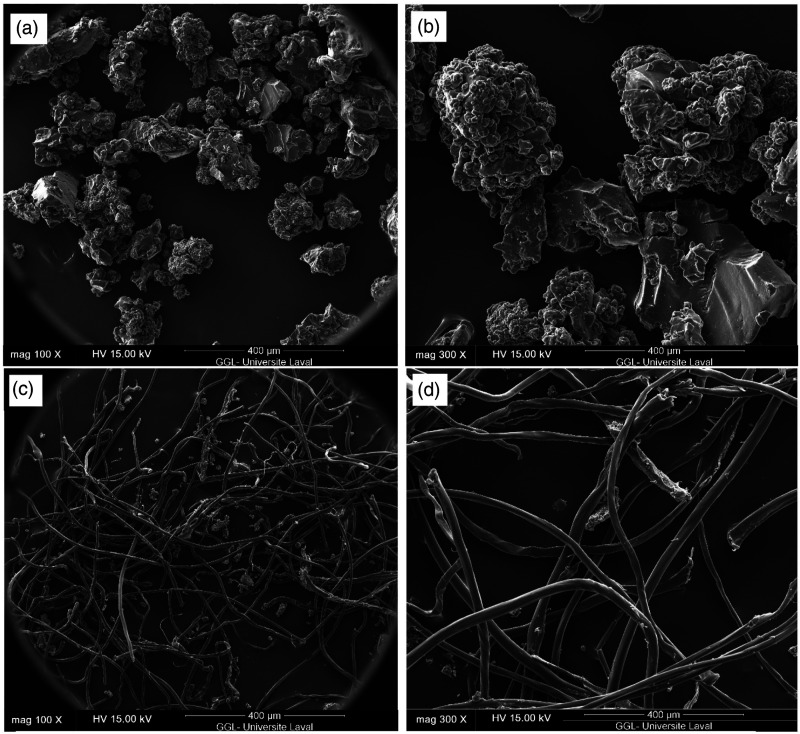
SEM micrographs of: (a and b) RR and (c and d) RTF at different magnifications. SEM: scanning electron microscope; RTF: recycled tire textile fiber.

Scanning electron microscope micrographs of RR particles and RTF are presented in Figure 4 at different magnifications. Several steps of waste tires grinding lead to a size reduction of both rubber/fibers and the heterogeneous nature of recycled materials making it difficult to obtain a specific size and distribution. Nevertheless, the SEM images show that for the material received, the RR particle size distribution is about 500 μm (Figure 4(a) and (b)), while the recycled fibers have a length and diameter of 1000–3000 μm and 20–30 μm (Figure 4(c) and (d)), respectively. The RR particles show irregular surfaces with cracks and different shapes of porous/smooth surfaces because of different types of tires and/or different grinding processes used for their production coupled with thermomechanical degradation during the regeneration step.

**Figure 5.**
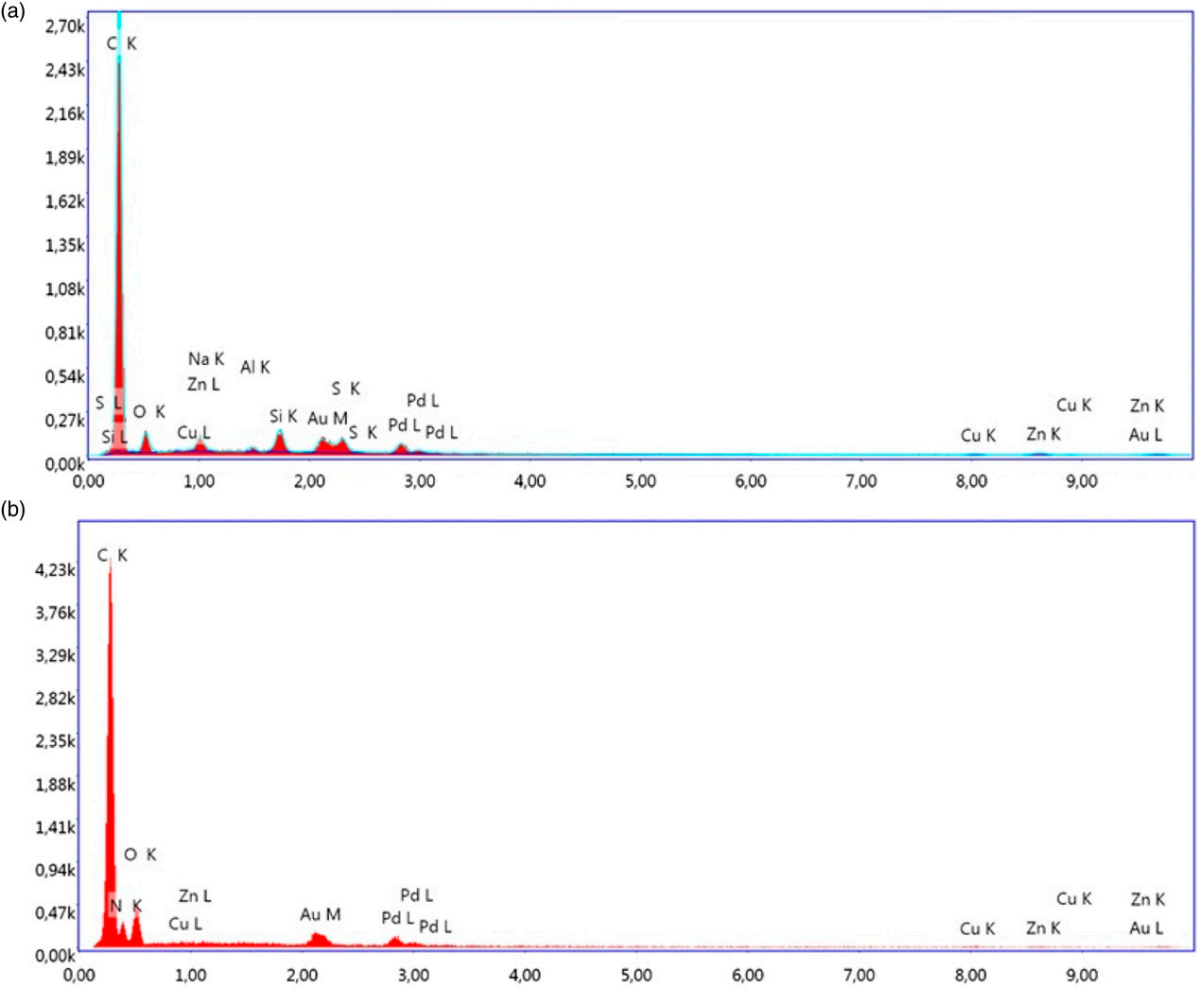
EDS spectra of: (A) RR and (B) RTF to show impurities. EDS: energy dispersive spectroscopy; RTF: recycled tire textile fiber.

Scanning electron microscope micrographs also show that the recycled rubber particles and fibers contain some impurities because of a wide variety of materials used in tires formulation. Energy dispersive spectroscopy analysis of RR (Figure 5(a)) and RTF (Figure 5(b)) indicates that typical impurities are mostly metal alloys and other additives (processing/vulcanization package) or polymeric materials.^36^ To get qualitative and quantitative analysis about these materials, the elemental compositions of RR and RTF are presented in Tables 3 and 4 respectively, in terms of weight and atomic percentage. The chemical analysis reveals the predominance of carbon and oxygen, while small amounts of S, Al, Si, Cu, and Zn are also detected. For example, sulfur and zinc oxides are part of the curing system used to crosslink the rubber, while aluminum silicates are reinforcing fillers leading to harder vulcanizates compared to calcium silicates. The presence of oxygen is associated to the additives and metal oxides.^37^

**Table 3.** Chemical analysis compositions of RR (EDS quantitative results).

Element	Weight (%)	Atomic (%)
C	83.13	92.28
O	5.09	4.24
Na	0.00	0.00
Al	0.39	0.19
Si	2.05	0.97
S	1.87	0.78
Cu	2.15	0.45
Zn	5.31	1.08

EDS: energy dispersive spectroscopy

**Table 4.** Chemical analysis compositions of RTF (EDS quantitative results).

Element	Weight (%)	Atomic (%)
C	66.92	72.66
N	16.59	15.45
O	13.96	11.38
Cu	1.37	0.28
Zn	1.16	0.23

EDS: energy dispersive spectroscopy; RTF: recycled tire textile fiber.

**Figure 6.**
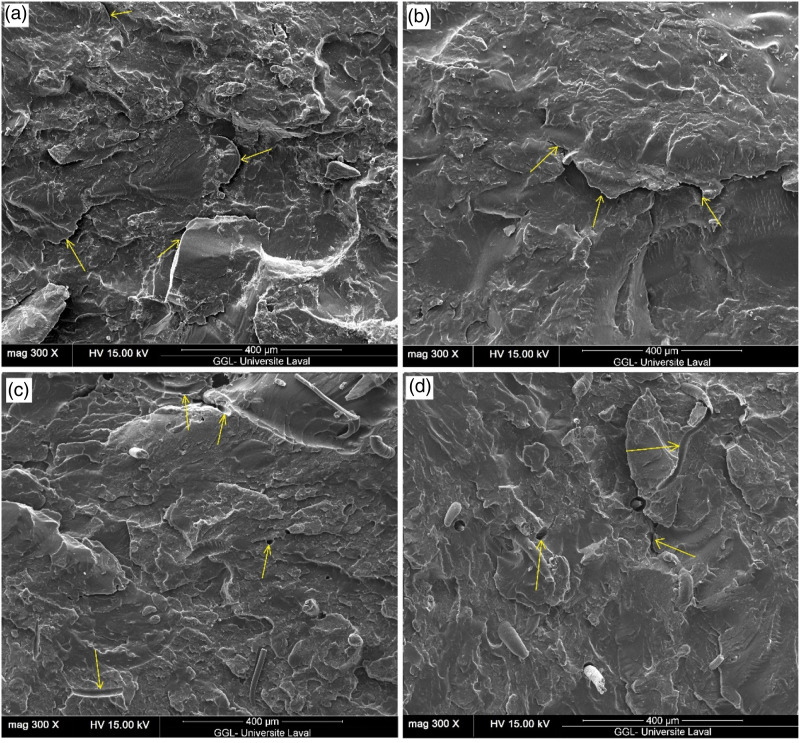
Scanning electron microscope micrographs of: (a) R60, (b) R80, (c) R45F and (d) R60F composites (arrows are used for easier identification of the failure phenomena).

Figure 6 presents typical SEM micrographs of cryogenically fractured cross-section surfaces of blends containing 60 and 80 wt.% of RR (Figure 6(a) and (b)) or RR/RTF mixture (Figure 6(c) and (d)). Micrographs of the compatibilized samples are also presented to compare the fracture behavior at the interface and general morphologies. As shown in Figure 6, large domains and protrusions of the dispersed phase indicate that the fillers have low affinity towards the rHDPE matrix due to incompatibility. In general, immiscible TPE blends present typical matrix/dispersed droplet-type morphology where large particle size of the dispersed domains (rubber phase) and sharp interface region between the crosslinked rubber and matrix indicate high interfacial tension between the components.^38^ Poorly bonded fillers to the matrix led to clean and smooth surface of R60 and R80 with voids around the fibers from debonding and/or rupture of the rubber particles, as well as easy pull-out of the dispersed rubber particles.^39,40^ This implies that the weak interface could not transfer the load from the matrix to the reinforcements and failure occurred at the interface.^40^ As shown in Figure 6(c) and (d), poor surface interaction between RTF and rHDPE (easy debonding and fiber pull-out from the matrix) in R45F and R60F leads to the formation of large voids/cracks around the fibers. This non-homogeneous morphology with poor adhesion between the phases (high interfacial tension) leads to low mechanical properties, especially as the number of defects increased with filler content.^28,29^

**Figure 7.**
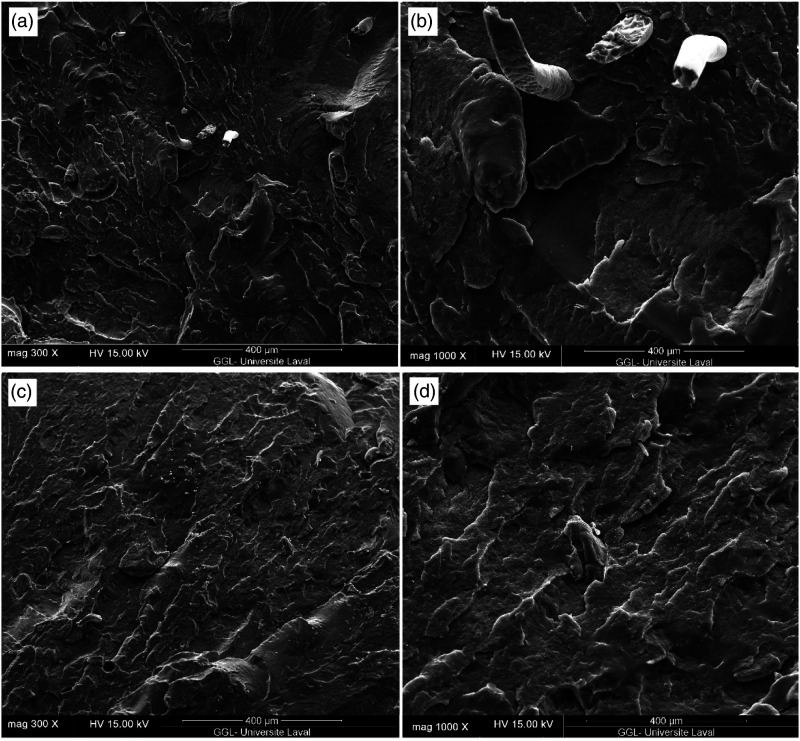
Scanning electron microscope micrographs of: (a and b) R45F* and (c and d) R60F* composites at different magnifications.

Scanning electron microscope micrographs are presented at different magnifications to get an idea of the interface quality of compatibilized composites (Figure 7). Phase morphology of multicomponent blends is determined by the interfacial interactions and compatibility between the phases which are known to control the compound properties.^38,39^ As shown in Figure 7, R45F* and R60F* show coarser fractured surface compared to their uncompatibilized counterparts (Figure 6(c) and (d)) as fewer gaps/voids at the filler/matrix interfaces can be seen (Figure 7(b) and (d)). This behavior is attributed to the improved interface quality and better fracture resistance. The presence of MAPE changed the morphology from a heterogeneous structure for uncompatibilized systems (Figure 6(c) and (d)) to a more homogeneous morphology for compatibilized ones (Figure 7(a) and (c)). Interactions between the compatibilizer and both RR and rHDPE result in stronger interfacial interaction (reduced interfacial tension) producing a good dispersion of the rubber phase in the matrix and a more homogeneous structure.^38^ Figure 7(b) and (d) also show that RR particles are completely embedded within the matrix as it is very difficult to distinguish them from the matrix on the fractured surfaces. Furthermore, much less gaps and defects are present which is ascribed to good rubber particles coverage (due to the masterbatch step used) by the compatibilizer to form molecular entanglement at the interface layer leading to better interfacial interaction.^29^ Improved compatibility between RR and compatibilizer is related to chemical bonds formed between the unsaturated C = C bonds on the rubber surface and the maleic anhydride group of MAPE.^41,42^ Contrary to R45F and R60F, no fiber pull-out is detected in R45F* and R60F*, so RTF are also well embedded in the matrix suggesting more affinity between the components (reduced surface energy), thereby increased failure resistance through effective load transfer can be expected.^22,25^ This special morphology is also expected to improve all the mechanical properties, especially the elongation at break and impact strength as described next.

### Mechanical (tension and flexion) properties

The effect of blend composition and compatibilizer addition on the mechanical and physical properties of the composites are presented in Table 5. Almost all the binary blends of thermoplastic resins filled with recycled rubbers (vulcanized structure) have very poor mechanical properties, especially low tensile strain at break and impact strength.^27^ This is attributed to very low entanglement between the crosslinked rubber particles and matrix (low compatibility) leading to the formation of voids around rubber particles (stress concentration points) facilitating crazing and interfacial debonding.^43^ Increasing the RR content decreases the tensile strength of all samples. For example, the tensile strength of R65 and R80 are 60% and 76% lower than neat rHDPE (19.0 MPa). Higher filler ratio (RR = soft phase) transformed into larger rubber agglomerates with high gel content (crosslinked) acting as stress concentration point at the interface of binary blends (polar and non-polar materials).^29^ Adding RTF to the rHDPE/RR compounds did not modify the tensile strength values showing poor fiber–matrix interaction. This can be related to the effect of reinforcing fibers (organic and inorganic) interfering the continuity of the matrix which indicates the prominent role of incompatibility between RR and rHDPE on the tensile properties.^16^ However, using the RR/MAPE masterbatch had a substantial effect on the tensile strength of compatibilized samples compared to their uncompatibilized counterparts. As shown in Table 5, the tensile strength of R60F* (8.8 MPa) is, respectively, 79% and 87% higher than that of R60F (4.9 MPa) and R80 (4.7 MPa). The addition of MAPE is shown to generate good blend compatibility and improved interfacial bonding promoting smooth stress transfer and hence improved tensile strength of the compatibilized samples. The interaction of the maleic anhydride group (MAPE) with the hydroxyl group on the carbon black surface or carboxyl groups of RR may be responsible for interfacial interaction between rubber and compatibilizer.^44,45^ It is also reported that possible reaction between zinc oxide (ZnO) as a component of RR (Table 3) with maleic anhydride (MA) during melt mixing can be responsible for the tensile strength improvement of compatibilized TPE blends.^46^

**Table 5.** Mechanical properties of the samples produced (see Table 2 for definition).

Sample	Tensile strength (MPa)	Young’s modulus (MPa)	Tensile strain at break (%)	Flexural modulus (MPa)
rHDPE	19.0 (0.3)	427.1 (14.9)	949.2 (26.4)	594.4 (11.3)
R35	13.0 (0.3)	191.2 (4.3)	38.1 (4.8)	384.1 (3.5)
R50	9.2 (0.3)	152.3 (3.2)	44.2 (7.2)	281.8 (5.4)
R65	7.7 (0.1)	99.3 (4.2)	56.7 (5.3)	189.4 (3.8)
R80	4.7 (0.4)	32.5 (5.4)	77.9 (8.6)	103.6 (4.7)
R15F	9.5 (0.1)	246.5 (6.1)	30.2 (6.1)	405.6 (2.1)
R30F	9.2 (0.3)	170.5 (6.6)	36.4 (4.9)	308.5 (3.8)
R45F	7.4 (0.2)	109.3 (4.7)	45.3 (6.4)	202.7 (3.5)
R60F	4.9 (0.1)	45.8 (5.2)	65.2 (5.7)	134.7 (2.9)
R15F*	13.2 (0.2)	277.3 (4.9)	64.5 (8.2)	437.9 (3.4)
R30F*	12.1 (0.2)	212.2 (5.3)	87.6 (7.9)	384.6 (4.5)
R45F*	9.8 (0.1)	126.5 (3.6)	138.2 (7.6)	262.5 (4.2)
R60F*	8.8 (0.4)	80.9 (4.5)	172.3 (8.3)	182.7 (5.1)

rHDPE: recycled high density polyethylene.

The increase in RR content from 35 wt.% to 80 wt.% showed a significant decreasing trend of Young’s modulus from 191.2 MPa to 32.5 MPa attributed to the substitution of the rigid thermoplastic resin with a soft rubber phase of low rigidity. It is well established that adding RR to thermoplastic resins decreases their tensile modulus because of the lower glass transition temperature of rubber compared to that of semi-crystalline plastic, so RR is in the rubbery state and has much lower modulus at room temperature.^38^ The introduction of RTF somewhat increased Young’s modulus because of the stiff nature of these short fibers and limited stress transfer from the matrix.^28^ For example, adding 20 wt.% RTF into the binary blends (rHDPE/RR) increased Young’s modulus of R35 and R80 from 191.2 MPa and 32.5 MPa–246.5 MPa (22%) and 45.8 MPa (40%) for R15F and R60F samples, respectively.

Flexural modulus results present a similar decreasing trend as tensile modulus by adding RR particles as R80 (103.6 MPa) show the lowest value compared to R60F (134.7 MPa) and R60F* (182.7 MPa) being 83% lower than rHDPE (594.4 MPa). But conversely, adding recycled fibers slightly increased the flexural modulus of all fiber-reinforced composites attributed to the replacement of rubber particle (RR) by stiffer reinforcements (RTF) in RR/RTF. This increasing trend is more noticeable at low RR content (R15F) since lower rubber concentration in TPE blends requires more stress for deformation.^18^ As shown in Table 5, the introduction of RTF (20 wt.%) increased the flexural modulus of R35 from 384.1 MPa to 405.6 MPa for R15F. Higher flexural modulus of R15F* (437.9 MPa) compared to R15F (405.6 MPa) and R35 (384.1 MPa) is obtained because the addition of a compatibilizer improved the interfacial adhesion between each phase (Figures 4, 6, and 7) similar to tensile properties. Also, it is claimed that maleated compatibilizers can promote surface crystallization to form a trans-crystalline layer around short fibers with higher rigidity and lower deformability contributing to much higher modulus.^47^

The introduction of RR particles into the matrix led to lower tensile elongation at break and the values are much less than that of rHDPE (949.2%). However, increasing the rubber content from 35 to 80 wt.% led to higher elongation at break of R80 by 104% (from 38.1 to 77.9%) due to the presence of a more elastic phase inducing higher deformation/elasticity.^27^ Also, the addition of a fixed concentration of rigid fibers (20 wt.% RTF) resulted in a further drop because of the lower volume fraction of the soft rubber phase replaced by rigid fibers (solid phase) with low elasticity and poor affinity with the matrix (Figure 6). Similarly, Moghaddamzadeh and Rodrigue^29^ observed a very low tensile strain at break (25%) of linear low-density polyethylene composites reinforced with recycled tire fibers (50 wt.%). A mixture of thermoplastic/rubber is considered as a TPE compound if it shows at least 100% deformation,^48^ so R45F* and R60F* are interesting compounds with elongation at break of 138.2% and 172.3%, respectively. The compatibilized samples exhibit the highest elongation at break among the samples studied in this work which is related to the rubber-toughening effect and enhanced interfacial adhesion due to MAPE which is in agreement with the morphological findings.^39^ It is well-documented that the compatibilizing effect of MAPE in TPE blends is attributed to the interaction between the MA group of maleated copolymers as a polar component with the natural rubber (NR) (the main component of RR) as a non-polar material.^38^

### Fracture analysis

**Figure 8.**
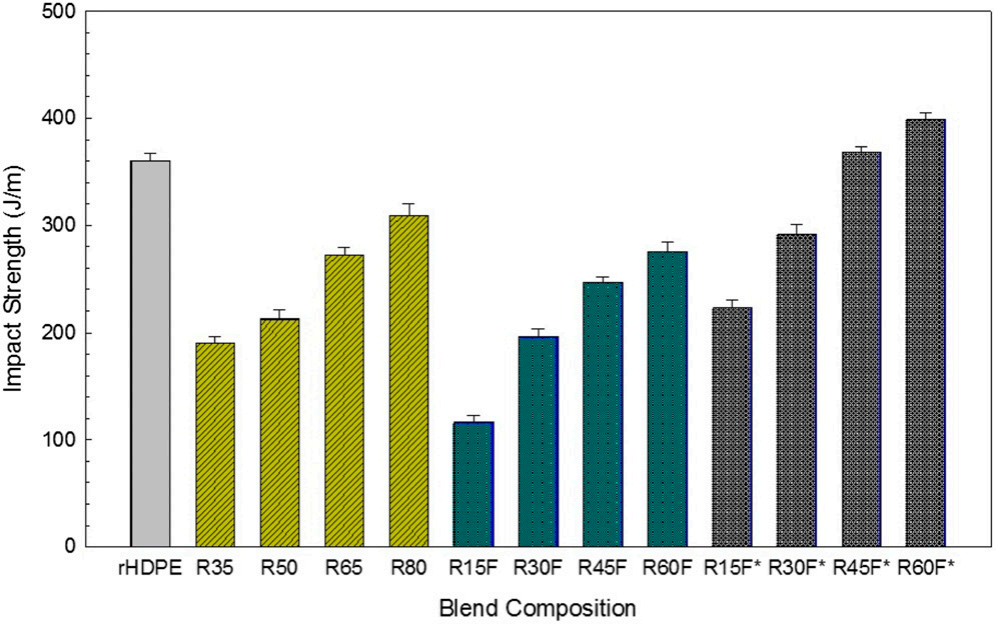
Impact strength of the samples produced (see Table 2 for definition).

The low impact strength (toughness) of short fiber-reinforced TPE, especially at low temperatures, limits the industrial application of such composites.^7,18^ Therefore, toughness improvement of these composites is of high importance. As shown in Figure 8, the toughness of R35 and R15F filled with only 35 wt.% of reinforcements (RR and RR/RTF (15/20)) are 48% and 68% lower than the impact strength of neat rHDPE (360 J/m). In a similar report, poor interfacial adhesion between filler and matrix decreased the impact strength of ethylene vinyl acetate from 72.3 J/m to 29.2 J/m (59%) upon the addition of 10 wt.% waste rubber crumbs (<200 μm).^49^ Despite the negative effect of filler content on toughness loss, further increase in recycled rubber content from 35 to 65 and 80 wt.% increased the toughness of R65 (272.5 J/m) and R80 (324.4 J/m) because of higher energy absorption through deformation of the rubbery particles retarding fracture phenomena.^50^ In agreement with Figure 8, Luna et al.^51^ reported toughness improvement in polystyrene (PS) composites by up to 77% with increasing recycled SBR content from 20 wt.% (37.5 J/m) to 50 wt.% (66.5 J/m). For rHDPE/RR/RTF blends, replacing the rubber phase with constant RTF content (20 wt.%) decreased the toughness of fiber-reinforced specimens as the impact strength of R45F and R60F are, respectively, 9% and 11% lower than R65 and R80, both having 65 and 80 wt.% of fillers. As discussed above, for fiber-reinforced TPE composites with low crack resistance, small microcracks, and sharp crack could easily propagate along with weak interfacial voids around rigid fibers resulting in reduced absorbed energy before sample failure.^52^ It should be noticed that the higher toughness of R60F (275.6 J/m) compared to R45F (246.5 J/m) is attributed to the higher content of regenerated rubber particles (lower crosslinked density) in R60F making the particles more deformable to absorb more energy and delay failure phenomena.^53^

The improved toughness upon increasing recycled filler content is at a cost of lower tensile strength and Young’s modulus (Table 5). Therefore, different attempts were made to produce a multiphase material with balanced toughness and tensile properties which can be obtained by the inclusion of an interfacial modifier to improve the compatibility of the blends.^18,20^ Surface coating of rubber crumbs (waste or virgin), using suitable block copolymer/compatibilizers which is compatible with the polyolefin matrix, forms a thick/soft interphase to improve bonding and promote smooth stress transfer between the RR and the matrix.^22^ Formela et al.^20^ observed that SBS, having partial miscibility with polyethylene and GTR, improved interfacial adhesion of LDPE/GTR blends by creating a strong interface between the matrix and rubber particles. As shown in Figure 8, a substantial increase in composites toughness is obtained by adding MAPE. The effect is more pronounced on the impact strength of R45F* (368.2 J/m) and R60F* (398.7 J/m) compared to R45F (246.5 J/m) and R60F (275.6 J/m). It can be assumed that MAPE surface coated RR seems to slow down crazing propagation through uniform filler dispersion in the matrix via thick interphase around RR particles reducing the stress concentration leading to more energy dissipated during crack growth (propagation).^54,55^ In a similar work, Kakroodi and Rodrigue^18^ reported about 81% higher toughness of PP–glass fiber composites (from 23.1 to 41.9 J/m) by adding 15% MAPP/EPDM compound because of improved interfacial adhesion as a result of the chemical similarity between EPDM and PP (propylene blocks) and strong bonding between C = C bonds in EPDM with MAPP. Also, impact modification of natural fiber-reinforced PP composites by the direct addition of MAPP coupling agent led to partially located MAPP at the interface of TPE blend with slightly improved toughness.^18^ It is well-documented that the efficiency of direct incorporation of compatibilizer depends on its localization at the interfacial zone, which subsequently would influence the homogeneity (filler dispersion) and interfacial strength which are controlled by the mixing strategy (component addition order).^39^ Based on tensile and impact properties, the strength of interfacial interactions increases with MAPE content, increasing the possibility of rubber encapsulation by more coupling agents contributing to better compatibility between the rubber and thermoplastic phases.^18^

### Physical (hardness and density) properties

In general, the hardness of a TPE compound is determined by the elastic modulus and crosslink density of the rubber phase (GTR).^6^ Table 6 shows that in spite of the presence of carbon black in recycled tire rubber, the hardness of the composites decreased with increasing RR content which is attributed to the soft nature of rubber particles with low rigidity.^40^ Also, the regeneration process results in a less crosslinked network (lower crosslink density) contributing to lower rigidity of the blends filled with RR.^27^ The variation of hardness with RTF addition follows a similar trend as the variation of tensile modulus (Table 5) and the addition of surface coated RR with MAPE did not modify this trend. For example, the introduction of 80 wt.% RR decreased the hardness (Shore D) of rHDPE from 66 to 39, while the hardness values are, respectively, 41.2 and 43.7 (Shore D) for R60F and R60F* filled with RR/RTF (60/20) and 60 wt.% RR/MAPE (70/30) masterbatch.

**Table 6.** Physical properties of the samples produced (see Table 2 for definition).

Sample	Hardness (Shore D)	Density (g/cm^3^)
rHDPE	66.0 (0.6)	0.986 (0.002)
R35	61.2 (0.4)	1.022 (0.001)
R50	54.3 (0.3)	1.039 (0.002)
R65	43.2 (0.7)	1.064 (0.003)
R80	39.0 (0.4)	1.093 (0.002)
R15F	63.4 (0.6)	1.052 (0.002)
R30F	55.1 (0.3)	1.095 (0.003)
R45F	47.1 (0.4)	1.112 (0.002)
R60F	41.2 (0.8)	1.129 (0.003)
R15F*	63.7 (0.5)	1.025 (0.003)
R30F*	56.8 (0.4)	1.069 (0.002)
R45F*	48.9 (0.4)	1.063 (0.003)
R60F*	43.7 (0.3)	1.084 (0.003)

rHDPE: recycled high density polyethylene.

Table 6 shows that the density increased due to the higher filler densities (RTF = 1.268 g/cm^3^ and RR = 1.184 g/cm^3^) compared to rHDPE (0.986 g/cm^3^) and MAPE (0.920 g/cm^3^). It should be noticed that fiber-reinforced rubberized composites filled with RR/RTF contain lower rubber content compared with RR filled composites. So the density of R60F (1.129 g/cm^3^) is higher than R80 (1.093 g/cm^3^), while R60F* has the lowest density (1.084 g/cm^3^) leading to superior specific mechanical properties (mechanical properties per unit of mass).^18^

### Thermal stability

**Figure 9.**
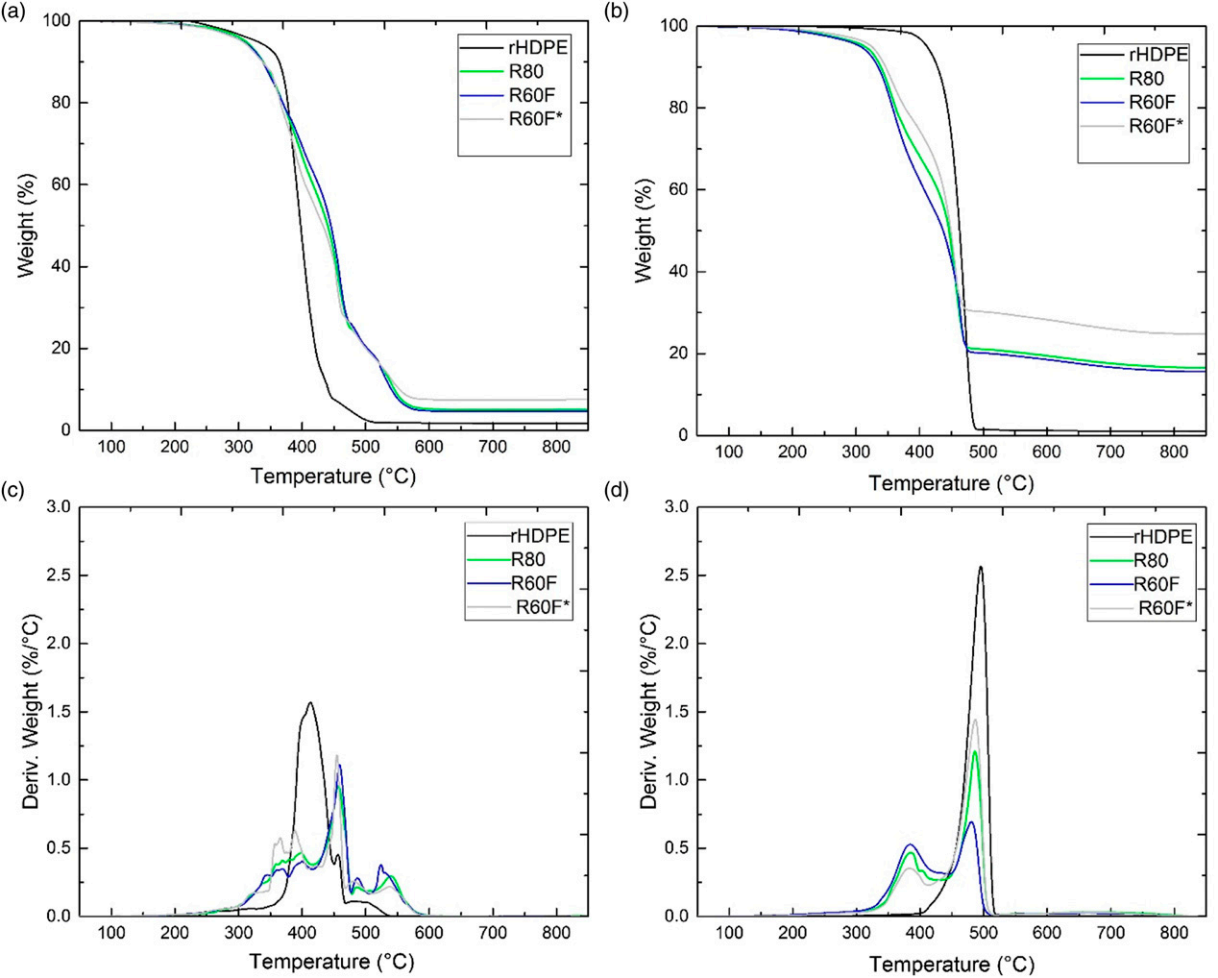
Weight and derivative curves as a function of temperature for rHDPE, R80, R60F and R60F* in: (a and c) air and (b and d) nitrogen (see Table 2 for definition). rHDPE: recycled high density polyethylene

Thermogravimetric analysis is an important characterization technique to determine the thermal stability of TPE since these materials are degraded during service life, as well as recycling (grinding) and regeneration processes which influence their long term properties.^40^ Table 7 presents an overview of the TGA results to compare the thermal and oxidative stabilities of the samples in terms of T_max_, which represents the temperature at which the rate of thermal degradation is at its peak evaluated from the derivative of the TGA curves (DTG), as well as T_10_ and T_50_ which represent the temperatures at which 10% and 50% of the initial mass disappeared, respectively. As shown in Table 7, the thermal stability of the neat materials can be classified in the order of (from the highest to the lowest thermal stability) MAPE > rHDPE > GTR > RTF. T_10_ for rHDPE and MAPE in air are 390°C and 394°C, respectively, compared to 303°C and 281°C for RR and RTF, respectively. So the introduction of both recycled rubber particles and tire fibers decreased the thermal stability of the thermoplastic resin as reported elsewhere.^7,21^ Table 7 also shows that T_10_ of R80 (341°C) and R60F (329°C) are lower than T_10_ of rHDPE (390°C) in air and T_10_ of these composites are also lower than that of rHDPE in nitrogen. Such low thermal stability can be ascribed to the presence of volatile material in the fillers such as processing oils, additives, and other compounds with low molar mass and/or low boiling temperature.^56^ Thermal decomposition temperatures are much higher in nitrogen compared to air (lower thermal stability in oxygen atmosphere) showing the effect of oxidation on the thermal decomposition of these compounds.^18^ In the case of compatibilized composites, R60F* shows 10% and 50% of initial mass loss at 352°C and 451°C in air, while T_10_ and T_50_ are at 371°C and 463°C in nitrogen. Higher T_10_ and T_50_ values suggest good compatibility of MAPE with rHDPE and RR associated with the good thermal stability of MAPE.^57^ The higher amount of residues can inhibit the degradation process of the undecomposed polymer as the out-diffusion of the volatile decomposition products is hindered by char content as a direct result of reduced permeability.^7^ For example, the residues of R60F* are 7.9% and 24.8% in air and nitrogen, respectively, which are higher than that of R80 and R0F composites. It is worth mentioning that Formela et al.^20^ reported higher thermal stability of LDPE/GTR (50/50) blends with the addition of SBS (compatibilizer) creating a soft interface around GTR particles improving interfacial adhesion and yielding higher residues for the compatibilized sample at 550°C by 39% (from 18.3 to 25.5 wt.%). For better comparison, the TGA and DTG curves of rHDPE, R80, R60F, and R60F* in air and nitrogen are shown in Figure 9. It is clear that the thermal decomposition of the TPE starts earlier than rHDPE attributed to the degradation of processing oils and additives at low temperature, as well as lower crosslink density of the generated rubber promoting its degradation at lower temperatures.^58^ Regardless of filler loading (RR or RR/RTF), the presence of recycled rubber particles increased the residues at 850°C compared to rHDPE. This observation can be related to the presence of minerals (such as carbon black and SiO_2_ usually around 30–35 wt.%) in the recycled tire formulation.^21^ The presence of the compatibilizer influenced the ultimate weight loss as R60F* compatibilized with 18 wt.% MAPE with strong molecular entanglements and interfacial adhesion between the phases showed the highest residues. Also, the carbon black content of tire rubber can adsorb low molecular weight volatile products formed during thermal degradation creating a barrier effect by producing a more tortuous path for these gases decreasing the ultimate weight loss.^58^ DTG curves show that the thermal degradation of TPE under air occurs as a multistep process related to rHDPE and MAPE degradation,^40^ decomposition of NR and synthetic rubber (SBR and/or BR),^58^ and carbon black leading to the formation of carbon dioxide.^59^ The difference between the residuals in air and nitrogen is related to an additional oxidation step of carbon black to CO_2_ around 540°C leading to lower value of residuals in air.^60^

**Table 7.** Decomposition temperatures (T_10_, T_50_, and T_max_) and residues of the samples produced (see Table 2 for definition).

Sample	T_max_ (°C)		T_10_ (°C)		T_50_ (°C)		Residues (wt.%)	
	Air	N_2_	Air	N_2_	Air	N_2_	Air	N_2_
rHDPE	423	491	390	425	423	479	1.1	1.6
MAPE	452	486	394	418	442	463	0.3	0.8
RR	341	417	303	342	466	435	7.4	32.7
RTF	338	394	281	305	391	423	4.5	11.4
R35	439	467	378	392	460	475	4.8	4.5
R50	440	475	364	378	457	470	4.2	8.8
R65	451	480	352	359	458	466	5.7	13.3
R80	459	489	341	343	450	466	6.6	15.9
R15F	437	464	365	379	458	472	3.4	5.6
R30F	443	473	355	368	456	469	4.5	7.9
R45F	450	478	341	356	455	465	6.1	12.6
R60F	456	486	329	347	450	461	6.4	15.5
R15F*	440	468	394	410	462	476	5.1	13.1
R30F*	445	477	375	400	460	471	5.8	16.8
R45F*	453	482	364	387	457	465	7.3	17.2
R60F*	466	489	352	371	451	463	7.9	24.8

MAPE: maleic anhydride grafted polyethylene; rHDPE: recycled high density polyethylene.

### Differential scanning calorimetry

The crystalline structure in TPE blends is of high importance as their mechanical properties are influenced by the matrix crystallinity, especially the impact strength of composites. Differential scanning calorimetry analysis was used to determine possible crystallinity changes of the matrix upon filler and compatibilizer addition. The melting (T_m_) and crystallization (T_c_) temperatures, melting enthalpy (ΔH_m_) and crystallinity degree (X) are summarized in Table 8. Earlier studies reported that the presence of crosslinked rubber only had a slight effect on the matrix microstructure because of the poor compatibility in binary blends.^61^ According to Table 8, the addition of RR and RTF resulted in small changes in T_m_ and T_c_ compared to rHDPE, and a slight increase in T_c_ of the compatibilized composites compared to the neat matrix. These results are attributed to the solid fillers dispersed in the semi-crystalline matrix improving heterogeneous nucleation. The lowest crystallization temperature of R60F* (117.9°C) among the compatibilized samples reflects a better filler encapsulation by MAPE since well covered and finely dispersed particles did not effectively improved heterogeneous nucleation.^39^ But increasing the filler (RR or RR/RTF) content led to a drop in ΔH_m_ which implies a perturbed crystallization by the presence of amorphous fillers. For example, R80 (26.1 J/g) and R60F (25.7 J/g) showed the lowest enthalpy of melting due to the lower content of crystallizable material (plastic phase).^21,23^ Variation in the crystallinity degree might influence the impact strength since higher level of crystallinity is known to reduce toughness.^62^ Restricted flowability of rubber particles (amorphous nature) increases the blend viscosity slowing down the diffusion of PE segments to crystallization sites (limited mobility of crystallizable chain segment) limiting the growth of lamellae on the crystalline side resulting in smaller crystalline phase and lower crystallinity.^20,23^ It is well-documented that the addition of virgin or recycled rubber into TPE contribute to lower chain regularity (restriction in mobility of the rHDPE chains) resulting in lower crystallinity level by limiting the growth of thick lamellas decreasing the crystallinity which is in agreement with lower tensile strength and modulus.^63,64^ It is also claimed that melt extrusion can lead to some crosslinking of the regenerated rubber particles (partially destroyed crosslinked network) which can served as local defects to interfere with the compact structure of the polymer chains, thus decreasing the crystallinity degree.^63^ Also, a small amount of short fibers (less than 10 wt.%) is reported to provide nucleation points to speed up the crystallization rate, but higher fiber loading (above 10 wt.%) prevents the spherulites from expanding in all direction, thus reducing crystallinity in agreement with our results (Table 8).^65^ Low crystallinity level of R80 (45.6%) suggests a decrease in the overall crystallinity with decreasing rHDPE content supports the decreasing trend of tensile strength and tensile modulus (Table 5) with increasing filler content (softer nature).^19^ Overall, the different blend compositions had negligible difference in their temperatures of melting and crystallization, as well as crystallinity degree which is in agreement with previous reports.^20^ According to the crystallinity and impact strength results, it can be concluded that the higher toughness (Figure 8) is mainly the results of the developed phase morphologies and interfacial interactions.

**Table 8.** Melting and crystallization temperatures with their corresponding enthalpy and crystallinity degree for the samples produced (see Table 2 for definition).

Sample code	T_m_ (°C)	T_c_ (°C)	ΔH_m_ (J/g)	X (%)
rHDPE	127.5	118.1	148.5	51.9
R35	126.6	118.1	94.1	50.6
R50	126.5	118.3	71.2	49.8
R65	127.0	117.8	46.3	46.2
R80	126.7	117.5	26.1	45.6
R15F	127.2	118.0	92.3	49.6
R30F	127.0	117.4	68.6	48.1
R45F	126.0	117.5	47.2	47.2
R60F	126.0	116.8	25.7	44.9
R15F*	127.1	118.6	103.4	52.2
R30F*	126.4	118.5	85.3	50.5
R45F*	127.0	118.3	69.4	49.1
R60F*	126.1	117.8	53.2	48.9

rHDPE: recycled high density polyethylene.

## Conclusion

This work proposed a simple approach to improve the impact strength of fiber-reinforced rubberized composites via surface coating of waste rubber particles with MAPE. TPE composites based on rHDPE/(RR/MAPE)/RTF reinforced with RR (35–80 wt.%) and RTF (20 wt.%) were investigated in terms of phase morphology, tensile/flexion properties, impact toughness, and thermal behavior. Despite a drop in tensile strength and Young’s modulus, the presence of RR particles improved the elongation at break of rHDPE/RR blends by up to 78% (R80) which was attributed to a higher rubber content (elastic phase) inducing higher deformation/elasticity. But substitution of the RR fraction by a RR/RTF mixture compensated these tensile/flexural losses because of the more rigid nature of RTF increasing the composites stiffness, while the impact strength decreased for the binary TPE compounds. A morphological characterization was used to confirm the level of blend interaction as surface coverage of RR particles with MAPE highly enhanced the interfacial adhesion between the fillers and rHDPE resulting in improved homogeneity (more uniform RR and RTF distribution). The presence of MAPE compatibilized the filler and matrix leading to improved tensile properties. The tensile strength of R80 was improved by 79% (from 4.7 MPa to 8.8 MPa) and the tensile strain at break was doubled (from 65.2% to 172.3%) for R60F*. Furthermore, significant impact strength improvement (up to 60%) was obtained after RR/MAPE masterbatch addition. This increased strength was more significant (up to 398.7 J/m) as the MAPE content increased up to 18 wt.% and also for samples with higher RR contents. It is concluded that improved compatibility between rHDPE and RR via MAPE formed stronger interface leading to reduced stress concentration around the fillers slowing down the fracture. Finally, the proposed processing step for encapsulation of the rubber phase by MAPE provided an efficient method for waste tire recycling (rubber and fibers) by producing toughened TPE composites with acceptable mechanical properties. The fiber-reinforced rubberized TPE composites studied in this paper have acceptable level of elasticity and toughness, thus having potential industrial applications such as sports equipment, automotive parts (bumper fascia, wiper blades, fender liners, sight shields, and stone deflectors), and construction industries (retrofit slabs, beams, signboards, and guardrails).
